# Cadaveric and Ultrasound Validation of Percutaneous Electrolysis Approaches at the Arcade of Frohse: A Potential Treatment for Radial Tunnel Syndrome

**DOI:** 10.3390/ijerph19042476

**Published:** 2022-02-21

**Authors:** Pedro Belón-Pérez, Laura Calderón-Díez, José Luis Sánchez-Sánchez, Miguel Robles-García, Gustavo Plaza-Manzano, César Fernández-de-las-Peñas

**Affiliations:** 1 Department of Physical Therapy, Real Madrid C.F., 28055 Madrid, Spain; pebelon@gmail.com; 2 Department of Physical Therapy, Universidad de Salamanca, 37008 Salamanca, Spain; lauca@usal.es (L.C.-D.); jlsanchez@usal.es (J.L.S.-S.); 3 Department of Anatomy and Histology, Faculty of Medicine, Universidad de Salamanca, 37008 Salamanca, Spain; mroblesgarcia@usal.es; 4 Department of Radiology, Rehabilitation and Physiotherapy, Universidad Complutense de Madrid, 28040 Madrid, Spain; gusplaza@ucm.es; 5 Instituto de Investigación Sanitaria del Hospital Clínico San Carlos, 28040 Madrid, Spain; 6 Department of Physical Therapy, Occupational Therapy, Physical Medicine and Rehabilitation, Universidad Rey Juan Carlos (URJC), 28922 Alcorcón, Spain; 7 Cátedra Institucional en Docencia, Clínica e Investigación en Fisioterapia: Terapia Manual, Punción Seca y Ejercicio Terapéutico, Universidad Rey Juan Carlos, 28922 Alcorcón, Spain

**Keywords:** Frohse arcade, radial nerve, supinator, cadaver, percutaneous electrolysis

## Abstract

Entrapment of the radial nerve at the arcade of Frohse could contribute to symptoms in patients with lateral epicondylalgia or radial tunnel syndrome. Our aim was to determine the validity of applying percutaneous electrolysis, targeting the supinator muscle at the Frohse’s arcade with ultrasound imaging and in a Thiel-embalmed cadaver model (not ultrasound-guiding). Percutaneous electrolysis targeting the supinator muscle was conducted in five healthy volunteers (ultrasound study) and three Thiel-embalmed cadaver forearms. Two approaches, one with the forearm supinated and other with the forearm pronated were conducted. The needle was inserted until the tip reached the interphase of both bellies of the supinator muscle. Accurate needle penetration of the supinator muscle was observed in 100% in both US-imaging and cadaveric studies. No neurovascular bundle of the radial-nerve deep branch was pierced in any insertion. The distance from the tip of the needle to the neurovascular bundle was 15.3 ± 0.6 mm with the forearm supinated, and 11.2 ± 0.6 mm with the forearm pronated. The results of the current study support that percutaneous electrolysis can properly target the supinator muscle with either the forearm in supination or in pronation. In fact, penetration of the neurovascular bundle was not observed in any approach when percutaneous needling electrolysis was performed by an experienced clinician.

## 1. Introduction

The radial nerve can be entrapped at different anatomical locations throughout its course. The radial nerve is one of the main peripheral nerve trunks of the upper extremity and arises from the posterior cord of the brachial plexus (nerve roots C5-T1) [1]. It crosses the axilla region, exits it inferiorly via the triangular interval, and descends down the arm, travelling laterally into the radial groove at the humerus [1]. To enter into the forearm, the radial nerve runs anterior to the lateral epicondyle, at the cubital fossa, before dividing on its superficial and deep nerve branches [1].

An important and underreported potential entrapment area of the deep branch of the radial nerve is when it passes between both layers of the supinator muscle, that is, the arcade of Frohse or supinator arch [2]. The arcade of Frohse is formed by a fibrous band between the superficial and deep layer of the supinator muscle, between which the deep branch of the radial nerve passes [2]. In fact, this area can exhibit different anatomical variations leading to a further radial nerve compression [3]. The compression of the posterior interosseous nerve (a continuation of the deep branch of the radial nerve) at the arcade of Frohse is called “radial tunnel syndrome” [4]. Individuals with radial tunnel syndrome exhibit symptoms, e.g., deep aching pain in the lateral elbow area and spreading dorsally and radially into the forearm during upper extremity activities, which are similar than those symptoms experienced by individuals with lateral epicondylalgia [4].

The first-line treatment of radial tunnel syndrome is usually conservative, however, there is a paucity in the literature regarding non-surgical treatment interventions for this condition [4]. The use of minimally invasive approaches has increased in recent years [5,6]. The rationale for applying minimally invasive interventions is that the increased tension and shortening of the muscular tissue, e.g., the supinator muscle, due the presence of taut bands, could increase the tension on the radial nerve and contribute to its entrapment [7]. In fact, a recent case report described the successful management of radial tunnel syndrome after applying dry needling, targeting the supinator muscle [8].

Anatomical landmarks represent the most common clinical method for applying needling interventions; however, if the needle pierces a nerve tissue, it could be damaged. Neuropraxia of the radial nerve has been described after application of dry needling at the junction of the middle and distal third of the humerus (where the radial nerve runs superficially) [9]. Accordingly, safety targeting of the muscle–nerve tissue is important. In the last decade, ultrasound imaging (US) has been used for better visualizing the radial nerve at the arcade of Frohse [10,11]. In fact, US is used for guiding the application of some needling interventions. For instance, Meng et al. reported that a US-guiding injection was a safe procedure for injecting the perineural sheath beneath the arcade of Frohse [12]. 

One needling intervention that is commonly US-guided is percutaneous electrolysis, an intervention consisting of the application of a galvanic electrical current throughout a filament needle. A recent meta-analysis found moderate evidence suggesting a positive effect of US-guided percutaneous electrolysis for pain and related disability in patients with musculoskeletal pain [13]. Further, an animal study has observed that the application of percutaneous electrolysis can release nerve tissue, i.e., the sciatic nerve, from a fibrous entrapment [14]. These authors proposed that percutaneous electrolysis would combine the mechanical effect of the needle and the galvanic current as a disruptive mechanism for the connective muscle tissue, thus freeing the nerve from the pressure of the surrounding tissue and improving the patient’s symptoms [14]. The aim of this study was to determine the validity of applying percutaneous electrolysis, targeting the supinator muscle at the arcade of Frohse with US imaging and also in a Thiel-embalmed cadaver model (not US-guiding).

## 2. Methods

### 2.1. Procedure

Five healthy volunteers participated in the US imaging study, whereas three Thiel-embalmed cadaver forearms (all males, all left-side) donated to the institutional university laboratory of the Universidad Autónoma de Madrid (Spain) were used for the cadaveric part. The forearm specimens were checked for the presence of any structural abnormality, that would influence the anatomical study. The procedure involving healthy participants was conducted following the Declaration of Helsinki and was approved by the Human Research Ethics Committee of the Universidad Rey Juan Carlos (Spain). Participants signed a written informed consent form prior to their inclusion.

### 2.2. Percutaneous Electrolysis Procedures

The US-guided percutaneous-needle intervention targeted the supinator muscle at the arcade of Frohse. The intervention was US-guided by using an Aplio a550 (CUS-A500) Canon^®^ device(Canon Medical System Europe BV, Spain Branch, Madrid, Spain)) equipped with a 14 MhZ superficial linear transducer (PTL-1005BT). The procedures were performed by a physical therapist with 15 years of experience in musculoskeletal ultrasound needling interventions. The ultrasound scan depth was set to 4.5 cm to ensure repeatability of the study in all subjects. In the short axis, the radial nerve was located on its point of bifurcation into the superficial and deep radial nerve branches. In that position, the deep branch of the radial nerve was identified as a relatively hypoechoic oval structure, surrounded by a rim of hyperechoic connective tissue deep in the brachioradialis muscle, and over the supinator muscle at the arcade of Frohse. 

**Figure 1:**
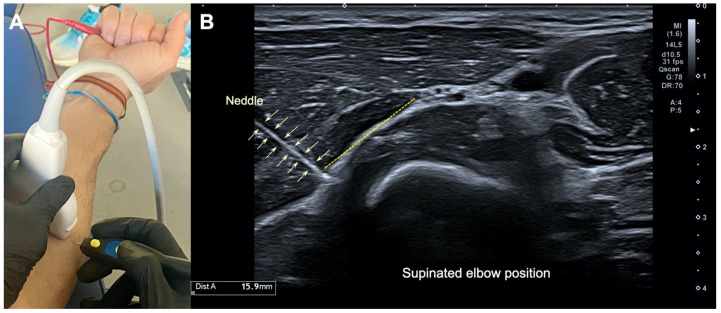
(**A**) Illustration of the percutaneous electrolysis approach with the elbow extended and supinated; (**B**) ultrasound imaging of the needle reaching the interphase of the supinator muscle at the arcade of Frohse, with the elbow extended and supinated.

**Figure 2:**
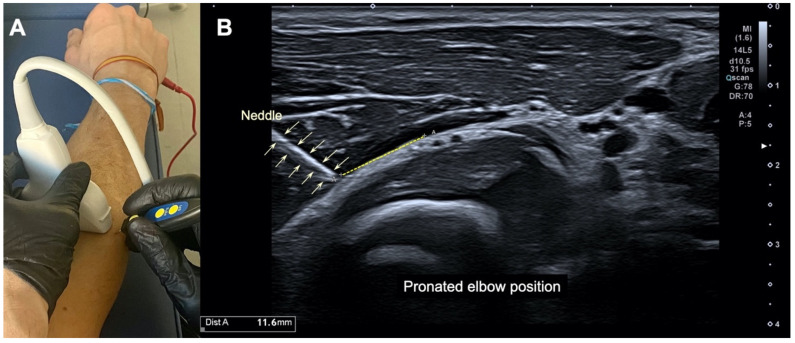
(**A**) Illustration of the percutaneous electrolysis approach with the elbow extended and pronated; (**B**) ultrasound imaging of the needle reaching the interphase of the supinator muscle at the arcade of Frohse, with the elbow extended and in pronation.

Two approaches were taken, the first one with the forearm in supination and the second one with the forearm in pronation, both with the elbow straight. In the supine position, a 25 × 0.3 mm filiform solid needle (AguPunt, Barcelona, Spain) was inserted from the lateral side of the forearm in the upper third of the radius, 4 cm distal to the lateral epicondyle (Figure 1). In the pronation position, a 25 × 0.3 mm filiform solid needle (AguPunt, Barcelona, Spain) was inserted at a 45° angle to the skin in the upper third of the radius, 4 cm distal to the lateral epicondyle (Figure 2). Both approaches were US-guided by the clinician to properly reach the interphase (beneath) of the supinator muscle at the arcade of Frohse (Figure 1B and Figure 2B). 

### 2.3. Anatomical Procedure over Thiel-embalmed Cadaver

The forearms were dissected in the long axis while the extremity was maintained with the elbow extended and in supination or pronation, respectively. Longitudinally, from 10 cm to the elbow to about 15 cm distal to the elbow, the skin and subcutaneous fascia tissue of the dorsal aspect of the forearm were removed; this allowed for visualization of the brachioradialis and deep branch of the radial nerve throughout the arcade of Frohse at the supinator muscle. The needle was inserted into the cadaver with all the tissues overlaid, and left in situ during the anatomical dissection to determine if the tip of the needle properly reached the supinator muscle. The cadaveric study was conducted without US-guiding.

## 3. Results

The US-imaging study revealed that the needle properly reached the interphase of the supinator muscle at the arcade of Frohse in all participants (accuracy of 100%) in both approaches, with the forearm supinated (Figure 1) and pronated (Figure 2). In the imaging, it can be seen that no neurovascular bundle was pierced during the needle procedure in any participant. The mean distance from the tip of the needle to the neurovascular bundle of the arcade of Frohse was 15.3 ± 0.6 mm with the forearm supinated (Figure 1A) and 11.2 ± 0.6 mm with the forearm pronated (Figure 2B).

**Figure 3:**
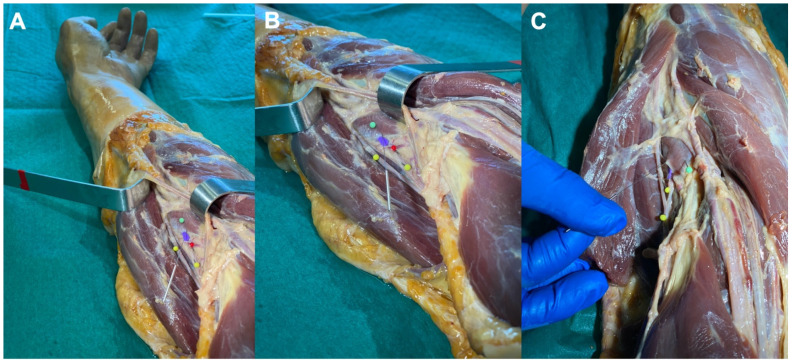
Scheme of the needling insertion with the elbow extended and supinated, showing that the needle reaches the supinator muscle: (**A**) general view; (**B**) detailed view; (**C**) rotation of the needle to confirm that the supinator muscle was properly reached. Colored corpse pins were inserted into the following structures: supinator muscle (green pin), radial recurrent artery (red pin), beneath the Frohse’s arcade (violet), and deep and superficial branches of the radial nerve (yellow pins).

**Figure 4:**
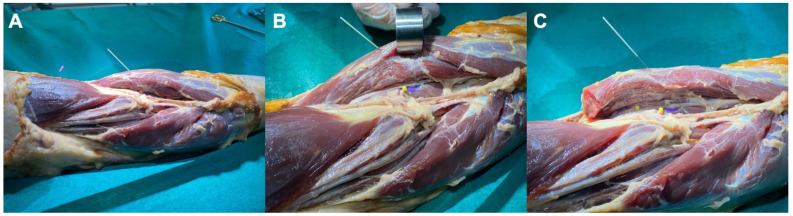
Scheme of the needling insertion with the elbow extended and in pronation, showing that the needle reaches the supinator muscle: (**A**) general view; (**B**) detailed view; (**C**) anatomical view with the extensor muscles disinserted. The figure shows the forearm in a horizontal position with the shoulder on the left side and the hand on the right side of the figure. Colored corpse pins were inserted into the following structures: beneath the Frohse’s arcade (violet), and deep and superficial branches of the radial nerve (yellow pins).

The anatomical study with the Thiel-embalmed cadaver revealed that the tip of the needle reached the supinator muscle belly in all forearm specimens (Figure 3), after passing throughout the brachioradialis muscle with the forearm supinated, and throughout the wrist extensor muscles (Figure 4) with the forearm pronated. No neurovascular bundle was pierced during the needle procedure in any specimen.

## 4. Discussion

The results of this study revealed that the application of percutaneous electrolysis, an intervention involving solid needling with a galvanic current, targeting the supinator muscle at the arcade of Frohse can be conducted with either the forearm in supination or in pronation. The results also show that the needle did not pierce the neurovascular bundle of the arcade of Frohse, although the distance to the deep branch of the radial nerve was slightly higher with the forearm in supination than in pronation. The deep branch of the radial nerve has a close anatomical relationship with the supinator muscle at the arcade of Frohse (through the interphase of the two muscular layers); however, anatomical variations in the relationship between the supinator muscle and the deep branch of the radial nerve at the arcade of Frohse can be present in some individuals [1,3].

Cadaveric studies permit the identification of therapeutic interventions with potential risk for neurovascular tissues that other methods do not permit [15]. This study found that needling insertion of the supinator muscle at Frohse arcade with both approaches provided enough space to avoid penetration of the neurovascular bundle, particularly if the procedure was US-guided. A recent cadaveric study reported distances of 8 mm from the tip of the needle to the different branches of the radial nerve during the application of dry needling with the forearm in pronation [16]. The distances observed in the current study during the US-guided intervention were higher than those reported in this previous study [16], supporting the potential improved safety of the procedure if US-guided. Additionally, the cadaveric study also revealed that the tip of the needle reached the radius bone in 80% of the specimens [16]. The application of US imaging allowed hitting the bone with the needle to be avoided in the current study. This could be relevant, since the tip of the needle suffers slight deformation when impacting a bone [17]. The application of a galvanic current during the application of percutaneous electrolysis increases the relevance of avoiding hitting the bone during the intervention.

Our results could be considered by clinicians applying percutaneous electrolysis for patients with lateral epicondylalgia [18] or radial tunnel syndrome to avoid injury to the radial nerve. In fact, clinicians should consider that the needle crosses the brachioradialis or the wrist extensor muscles before reaching the supinator muscle at the arcade of Frohse. 

Finally, some limitations should be recognized. First, US imaging and anatomical dissections were conducted in a small number of individuals and specimens, respectively. No data for gender differences in needle placement were able to be collected. Similarly, anthropometric forearm and wrist data could influence the observed distances to the radial neurovascular bundle. Second, we used a standardized anatomical landmark for targeting the supinator muscle at the arcade of Frohse in both approaches; accordingly, data should be considered for this approached point. Third, all needling insertions were conducted just once by an experienced clinician. We do not know the safety and accuracy of this needling procedure when applied by a novice clinician, or the reliability of either approach.

## 5. Conclusions

The results of the current study support that percutaneous electrolysis can properly target the supinator muscle with either the forearm in supination or in pronation. In fact, penetration of the neurovascular bundle of the deep branch of the radial nerve was not observed in any needle approach when performed by an experienced clinician.

## Data Availability

All data are included in the current version of the paper.
